# When Anger Motivates: Approach States Selectively Influence Running Performance

**DOI:** 10.3389/fpsyg.2020.01663

**Published:** 2020-08-05

**Authors:** Grace E. Giles, Carlene A. Horner, Eric Anderson, Grace M. Elliott, Tad T. Brunyé

**Affiliations:** ^1^U.S. Army Combat Capabilities Development Command Soldier Center, Natick, MA, United States; ^2^Center for Applied Brain and Cognitive Sciences, School of Engineering, Tufts University, Medford, MA, United States; ^3^Center for Outcomes Research and Evaluation, Maine Medical Center Research Institute, Portland, ME, United States

**Keywords:** exercise, motivation, approach, avoidance, emotion

## Abstract

Emotional states are thought to influence athletic performance. Emotions characterized by high arousal enhance exercise performance. Extant research has focused on the valence and arousal dimensions of emotions, but not whether the motivational dimension (the extent to which the emotion engenders approach or avoidance behaviors) influences exercise performance. Two studies aimed to determine whether films and music chosen to induce approach- (i.e., anger), avoidance- (i.e., fear), and neutral-oriented emotions would successfully induce their intended emotional states (Study 1) and whether anger and fear emotion inductions would influence 2-mile time trial performance (Study 2). In Study 1, the films and music successfully induced their intended emotions. In Study 2, run time and perceived level of exertion did not differ between emotions across all participants or among faster running participants per a median split. However, among slower running participants, the anger induction increased the 2-mile running speed relative to the neutral induction. These findings suggest that emotions eliciting approach-related motivational states may improve exercise performance, particularly in slower runners.

## Introduction

Emotional states are thought to influence athletic performance. Multiple retrospective, cross-sectional studies suggest that precompetitive emotional states are associated with perceived athletic performance. For instance, emotional states perceived as under control were thought to facilitate swimming performance, whereas those perceived as out of control were thought to hinder performance (Hanton and Connaughton, 2002). Certain pleasant emotions such as interest and enjoyment and certain unpleasant emotions such as sadness, guilt, and self-hostility predicted perceived athletic performance (Cerin, 2003). Further, precompetitive unpleasant feelings of anger, confusion, depression, fatigue, and tension were associated with greater dysfunctional than optimal performance, whereas pleasant feelings of calmness, happiness, and vigor were associated with greater optimal than dysfunctional performance (Lane et al., 2010).

Within experimental work evaluating the influence of emotion on anaerobic exercise performance, relatively high-tempo “arousing” music during a 10-min warm-up increased peak anaerobic power (Eliakim et al., 2007; Jarraya et al., 2012). Similarly, “stimulating” music increased grip strength relative to “sedative” music (Pearce, 1981; Karageorghis et al., 1996). Other work has manipulated mood using imagery scripts and emotional pictures. Peak force produced during isometric leg extensions was greater following an imagery script intended to induce anger than those intended to induce happiness or a neutral emotional state (Woodman et al., 2009). Negative and positive pictures increased force strength on a handgrip exercise relative to neutral pictures (Schmidt et al., 2009).

Such results may translate to aerobic exercise performance as well, as “arousing” music intended to “emotionally charge up” participants enhanced 60-m dash performance among collegiate track and field sprinters (Hall and Erickson, 1995). Other research has found that music perceived as motivating benefitted self-reported running performance (Lane et al., 2011). Indeed, whether emotional states are perceived as functional or not functional may drive their impact on performance. According to the individual zones of optimal functioning (IZOF) model, emotions can be pleasant and unpleasant as well as functionally optimal or dysfunctional (Ruiz et al., 2017). For instance, experiencing anger and anxiety may alter performance in optimal and suboptimal ways between individuals and sports (Ruiz and Hanin, 2011). Thus, the extant literature generally suggests that arousing emotions, such as anxiety or anger, enhance exercise performance, but these effects differ across individuals. However, research has focused on relatively short-duration, often-anaerobic exercise performance, and the valence and arousal dimensions of emotions. To our knowledge, no study has examined the extent to which the motivational dimension of emotion influences aerobic exercise performance.

Emotional experience is thought to be organized by two distinct motivational systems: approach and avoidance (Carver, 2006). Anger and fear are two negative-valence, high-arousal emotions that occur in response to threat, but differ in the motivation they trigger to approach or avoid the threat (Newhagen, 1998). In other words, they are similar in valence and arousal, but differ in the motivational response they engender. Anger is appraised as a personal offense (Lazarus, 2000). It is approach-oriented in that it motivates an individual to attack the threat (Carver and Harmon-Jones, 2009). Fear is appraised as an impending danger (Lazarus, 2000) and is thus avoidance-oriented in that it motivates an individual to flee from threat (Lazarus, 2001; Lee and Lang, 2009).

Different motivational states are known to alter action dispositions and promote varied behavioral drives (Carver and Scheier, 1990; Davidson et al., 1990; Bradley, 2000). Traditionally, positive affect was associated with an approach-related drive, whereas negative affect was associated with an avoidance-related drive. However, there are some exceptions to this dissociation, namely, that within negatively valenced emotional states, fear can promote avoidance whereas anger can promote approach (Harmon-Jones and Allen, 1998; Harmon-Jones, 2003; Carver and Harmon-Jones, 2009). Inducing approach- versus avoidance-oriented motivational states can alter several aspects of cognitive and physical performance. For instance, inducing approach- or avoidance-related motivational states can improve cognitive control (Savine et al., 2010), and inducing anger can accelerate approach-related joint-specific and gross motor behaviors (Marsh et al., 2005; Mayan and Meiran, 2011). Regarding joint-specific behavior, researchers showed participants approach- or avoidance-inducing stimuli and found that approach states facilitated arm extension (reaching toward) and avoidance facilitated arm flexion (pulling away) (Marsh et al., 2005). Regarding gross motor behavior, researchers induced anxiety (avoidance) or anger (approach) and found that participants were faster to initiate a step forward in the approach versus avoidance and control conditions (Mayan and Meiran, 2011). Some believe that the priming of approach-oriented behavior under conditions of anger is due to a disruption of a broader need to satisfy goals and an increased drive to behaviorally “push forward” and pursue those goals (Frijda, 1988). However, it is unknown whether any such effects of motivational states are robust enough to alter whole-body aerobic exercise performance.

The present studies fulfill two primary objectives in relation to this extant research. First, we seek to extend research examining motivational state influences on behavior, better isolating the influence of motivational states (in addition to arousal and valence effects) on behaviors increasingly reflective of real-world actions (i.e., athletic performance). Second, we seek to extend research showing the influence of motivational states on single-joint and step onset-related behaviors by examining whole-body aerobic exercise. To accomplish these objectives, we conducted two studies. The first aimed to determine whether films and music chosen to induce anger, fear, and neutral emotions would successfully induce those emotions (Study 1), and the second aimed to determine whether anger- and fear-associated motivational state inductions would influence aerobic exercise (in the form of 2-mile time trial performance) relative to a neutral motivational state induction (Study 2).

## Study 1

### Methods

#### Participants

Thirty-four individuals (20 women; age 18–50 years) participated for monetary compensation of $20 USD per hour (see Table 1). Informed consent was obtained from all individual participants included in the study, and both the Tufts University Institutional Review Board and the Army Human Research Protections Office approved all procedures.

**TABLE 1:** Study 1 sample characteristics (*n* = 34).

	Mean	SD	Minimum	Maximum
Age	25.5	7.3	18	50
BMI	25.0	6.0	19.4	41.2
Godin Leisure Time Questionnaire	50.8	28.7	0	107
BAS Drive	8.5	2.5	5	15
BAS Fun Seeking	8.2	2.3	4	14
BAS Reward Responsiveness	7.3	2.6	5	15
BIS	13.4	3.8	8	21

*BMI, body mass index; BAS, Behavioral Activation System; BIS, Behavioral Inhibition System.*

#### Research Design

Study 1 used a repeated measures design, with motivational state induction (approach, avoidance, neutral) as the within-participants factor. Sample size estimation was based on effect sizes from Lane et al. (2010) who found that emotional states were associated with optimal and dysfunctional performance (η_*p*_^2^ = 0.39). Using G^∗^Power (Faul et al., 2007) the necessary sample size was estimated to be 21 with an alpha level of *p* = 0.05, a power of 0.95, using repeated measures analysis of variance (ANOVA) with two degrees of freedom (Lane et al., 2010).

#### Measures

The Godin Leisure Time Exercise Questionnaire and Behavioral Approach System/Behavioral Avoidance System (BAS/BIS) were administered at the start of the study to capture sample characteristics. The Discrete Emotions Questionnaire (DEQ) was administered at multiple time points during each of the three experimental sessions.

##### Godin leisure time questionnaire

The Godin Leisure Time Exercise Questionnaire quantified participants’ activity level and asked participants the number of times they engaged in strenuous, moderate, and light exercise for at least 15 min over an average week (Godin and Shephard, 1985). Weekly frequencies of strenuous, moderate, and light activities are then multiplied by nine, five, and three, respectively, to calculate a weekly leisure activity score. Individuals who score at least 24 are considered active, and those who score less than 14 are considered inactive (Godin, 2011). The Godin Leisure Time Questionnaire has been shown to be valid in classifying individuals as active and insufficiently active (Amireault and Godin, 2015).

##### Behavioral approach system/behavioral avoidance system (BAS/BIS) scales

The BAS/BIS scales evaluated individual differences in approach and avoidance motivation systems (Carver and White, 1994). The BIS/BAS scales consist of a single Inhibition scale and Approach subscales of Reward Responsiveness, Drive, and Fun Seeking. Participants are asked to respond to 24 items by indicating on a four-point scale how true each statement is from them (e.g., “I crave excitement and new sensations” and “I worry about making mistakes”). The BIS/BAS show adequate internal consistency reliability (α = 0.66–0.76) (Carver and White, 1994) and test–retest reliability (ICC = 0.41–0.42) (Schneider et al., 2016).

##### The discrete emotions questionnaire (DEQ)

The anger and fear subscales of the DEQ measured participants’ self-reported feelings of anger and fear. Participants rated the extent to which they experienced emotions such as “anger,” “rage,” and “mad” (anger subscale) and “scared,” “panic,” and “fear” (fear subscale) on a scale ranging from “Not at all” (1) to “An extreme amount” (7). These subscales show adequate internal consistency reliability (α ≥ 0.92) and sensitivity to detect changes in discrete emotions (Harmon-Jones et al., 2016).

##### Motivational state induction

Approach and avoidance motivational states were operationalized by experimentally inducing feelings of anger and fear, based on evidence that anger and fear are both negative-valence, high-arousal emotions that occur in response to threat, but differ in the motivation they generate to approach (anger) or avoid (fear) the threat (Newhagen, 1998). To do so, participants viewed a series of films previously validated to engender neutral, fearful, or angry feelings (Betz et al., 2015). Four film clips (11 min, 17 s) used to induce anger included news stories of sexual and verbal harassment and gang violence, and a movie featuring verbal child abuse. Three film clips (11 min, 47 s) used to induce fear included individuals being chased by unseen threats and unexpected appearances of ghost-like individuals. Three film clips (9 min, 35 s) used to induce a neutral emotional state included a lesson on descriptive statistics, office discussions, and a bowling competition. To extend the motivational state induction, participants listened to music that was also validated to evoke neutral, fearful, or angry feelings (Ford et al., 2010). The music consisted of 5-min instrumental pieces played on repeat.

#### Procedure

First, informed consent was obtained from all individual participants. Participants then completed the Godin Leisure Time Questionnaire and the BAS/BIS scales. Participants then completed a baseline DEQ. Participants watched the neutral-, anger-, or fear-inducing films. They then completed a DEQ immediately following the films. Next, the neutral-, anger-, or fear-inducing music was started, which continued for the next 30 min. During this time, participants completed the DEQ every 5 min. The three test sessions were spaced at least 1 day apart and were identical except for the motivational state induction. Following the third test session, participants were fully debriefed and compensated for their participation.

#### Statistical Methods

The DEQ was analyzed using a repeated measures ANOVA with motivational state induction (Neutral, Anger, Fear), and Time (Pre-Induction, Minutes 0, 5, 10, 15, 20, 25, 30 Post-Induction) as within-participant factors.

An effect was deemed statistically significant if the likelihood of its occurrence by chance was *p* < 0.05. When sphericity was violated, Greenhouse–Geisser corrected *p*-values were used. When an ANOVA yielded a significant main effect, *post hoc* tests using the Bonferroni correction were conducted. Effect sizes are reported as η^2^ for ANOVAs and Cohen’s *dz* for *t*-tests (Lakens, 2013). All statistical analyses described above were performed using SPSS 21.0.

### Results

Analysis of self-reported anger showed main effects of motivational state induction, *F*(2,66) = 17.671, *p* < 0.001 (η^2^ = 0.261), and time, *F*(7,231) = 7.840, *p* < 0.001 (η^2^ = 0.057), which were qualified by a motivational state induction by time interaction, *F*(14,462) = 8.293, *p* < 0.001 (η^2^ = 0.111); see Table 2. Follow-up tests showed that self-reported anger was greater during the anger than neutral or fear induction immediately through 25 min following the films (*p-*values < 0.01). Thirty minutes following the films, self-reported anger was greater during the anger than neutral induction (*p* = 0.003), but did not differ between the anger and fear induction (*p* = 0.081). Self-reported anger did not differ across motivational state inductions before inductions (*p* = 0.61).

**TABLE 2:** Study 1 self-reported anger and fear before, during, and after anger, fear, and neutral motivational state inductions (*n* = 34).

		Self-reported anger				Self-reported fear			
		Mean	SEM	Min	Max	Mean	SEM	Min	Max
Anger induction film	Pre-induction	1.16	0.07	1.00	2.75	1.13	0.05	1.00	2.00
	0 min Post-induction	3.49	0.28	1.00	7.00	1.88	0.19	1.00	5.00
	5 min Post-induction	2.03	0.22	1.00	5.33	1.55	0.18	1.00	5.75
	10 min Post-induction	2.08	0.28	1.00	7.00	1.51	0.19	1.00	5.75
	15 min Post-induction	2.18	0.32	1.00	6.67	1.61	0.21	1.00	6.00
	20 min Post-induction	2.22	0.32	1.00	7.00	1.60	0.22	1.00	6.00
	25 min Post-induction	2.25	0.33	1.00	7.00	1.63	0.20	1.00	6.25
	30 min Post-induction	2.27	0.33	1.00	7.00	1.68	0.24	1.00	6.00
Fear induction film	Pre-induction	1.28	0.14	1.00	5.25	1.17	0.10	1.00	3.75
	0 min Post-induction	1.39	0.11	1.00	3.33	3.25	0.32	1.00	7.00
	5 min Post-induction	1.30	0.15	1.00	6.00	2.04	0.22	1.00	5.75
	10 min Post-induction	1.53	0.20	1.00	6.67	1.85	0.20	1.00	5.00
	15 min Post-induction	1.38	0.15	1.00	5.67	1.65	0.19	1.00	5.75
	20 min Post-induction	1.35	0.14	1.00	5.00	1.43	0.16	1.00	4.75
	25 min Post-induction	1.47	0.19	1.00	6.33	1.43	0.20	1.00	6.75
	30 min Post-induction	1.70	0.25	1.00	7.00	1.41	0.17	1.00	4.75
Neutral induction film	pre-induction	1.29	0.12	1.00	4.67	1.25	0.10	1.00	3.25
	0 min Post-induction	1.15	0.10	1.00	4.00	1.07	0.03	1.00	1.75
	5 min Post-induction	1.10	0.05	1.00	2.33	1.04	0.03	1.00	1.75
	10 min Post-induction	1.10	0.06	1.00	2.67	1.07	0.04	1.00	1.75
	15 min Post-induction	1.14	0.07	1.00	2.67	1.07	0.03	1.00	1.75
	20 min Post-induction	1.23	0.11	1.00	4.33	1.05	0.03	1.00	2.00
	25 min Post-induction	1.16	0.07	1.00	2.75	1.10	0.04	1.00	2.00
	30 min Post-induction	1.27	0.10	1.00	3.25	1.07	0.03	1.00	1.75

*SEM, standard error of the mean.*

Analysis of self-reported fear showed main effects of motivational state induction, *F*(2,66) = 13.534, *p* < 0.001 (η^2^ = 0.113), and time, *F*(7,231) = 14.874, *p* < 0.001 (η^2^ = 0.080), which were qualified by a motivational state induction by time interaction, *F*(14,462) = 14.839, *p* < 0.001 (η^2^ = 0.109). Follow-up tests showed that self-reported fear was greater during the fear than neutral or anger induction immediately through 10 min following the films (*p*-values < 0.001). Self-reported fear was greater during the fear than neutral condition 15, 20, and 30 min following the films (*p-*values < 0.05), but did not differ between fear and anger at these time points (*p*-values > 0.15).

### Discussion

As expected, the anger- and fear-inducing films and music increased self-reported anger and fear, respectively. Specifically, anger-inducing films and music resulted in higher self-reported anger than fear for at least 25 min following viewing the films. Similarly, fear-inducing films and music resulted in higher self-reported fear than anger for at least 10 min following viewing the films. Both inductions increased their intended emotions relative to neutral for the entire 30 min. Such findings suggest that the chosen films and music will similarly induce anger and fear states throughout the 2-mile time trial in Study 2, given that the median pace in 5-km races is 9:16 min per mile in men and 11:15 min per mile in women (Douglas and Fuehrer, 2014).

## Study 2

### Methods

#### Participants

Thirty-four individuals (19 women; age 18–35 years) participated for monetary compensation of US$150 (see Table 3). All participants exercised for at least 30 min of moderate-intensity cardiorespiratory exercise 5 or more days per week, or at least 20 min of vigorous-intensity cardiorespiratory exercise for at least 3 days per week (Garber et al., 2011), and ran at least 2 consecutive miles in 2 weeks prior to their participation. Participants also had healthy vision and hearing, were not pregnant or nursing, and did not have any contraindications to exercise such as orthopedic injuries, heart or lung problems, or asthma. Informed consent was obtained from all individual participants included in the study, and both the United States Army Research, Development, and Engineering Command and the Tufts University Institutional Review Board approved all procedures.

**TABLE 3:** Study 2 sample characteristics (*n* = 34).

	Mean	SD	Minimum	Maximum
Age	24.9	4.8	18	34
BMI	21.5	2.3	18.0	29.3
Cardiorespiratory exercise (minutes/week)	284.8	192.4	60	1086
Longest weekly run (miles)	6.5	5.6	2	33
Godin Leisure Time Questionnaire	68.8	17.4	38	101
BAS Drive	8.1	2.3	4	14
BAS Fun Seeking	7.3	2.1	4	12
BAS Reward Responsiveness	7.3	1.9	5	12
BIS	14.4	3.2	8	20

*BMI, body mass index; BAS, Behavioral Activation System; BIS, Behavioral Inhibition System.*

#### Research Design

Study 2 used a repeated measures design, with motivational state induction (approach, avoidance, neutral) and Time (Pre-Induction, Post-Induction, Mile 1, Post-Cool-down, Post-Recovery) as within-participant factors. Sample size estimation was identical to Experiment 1, with the necessary sample size of 21 (Lane et al., 2010).

#### Measures

As in Study 1, the Godin Leisure Time Exercise Questionnaire and BAS/BIS were administered at the start of the study to capture sample characteristics. The DEQ and Borg Rated Perceived Exertion (RPE) Scale were before, during, and after exercise to measure endurance exercise experience. Approach and avoidance motivational states were experimentally induced using the same films and music as Study 1.

##### Borg rated perceived exertion (RPE) scale

This commonly used one-item self-report scale measures perceived physical exertion (Borg, 1982). Participants were told, “Choose the number that best describes your level of exertion,” on a scale ranging from “no exertion at all” (6) to “maximal exertion” (20).

##### Two-mile time trial

Participants first warmed up for 5 min by walking at 2.5 miles per hour (MPH). After 5 min, they ran for 2 miles at a self-selected pace intended to mimic race conditions. After 2 miles, they cooled down by walking at 2.5 MPH for 5 min and then sat quietly for a 15-minute recovery period. The 2-mile time trial was chosen as it is a common test of physical fitness (e.g., it is a component of the Army Physical Fitness test; Knapik and East, 2014) and is highly correlated with maximal oxygen consumption, a test of aerobic fitness capacity (Mello et al., 1987). Participants were given the following instructions before beginning the time trial:

“You will now complete a 2-mile time trial. You will run for 2 miles. Your goal is to complete the 2 miles as quickly as possible. You should also run within a pace that you feel you can safely maintain, that is, we do not want you to run at a pace that may cause you to trip or in any way injure yourself. Your cumulative distance will be displayed on the treadmill, but your pace and time will be hidden. You will be asked to complete two questionnaires while running, when you finish the first mile, which will ask about your level of physical exertion and emotions. You will answer these questions using the keypad on the treadmill. Do you have any questions?”

#### Procedure

**FIGURE 1:**
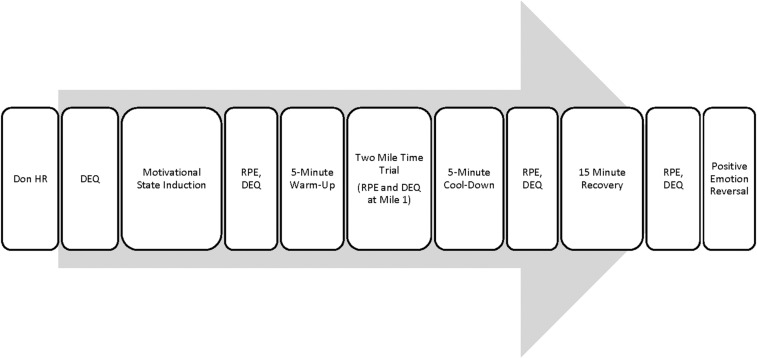
Schematic of Study 2 schedule.

First, informed consent was obtained from all individual participants. Participants were then screened for eligibility. If selected to participate, they completed the Godin Leisure Time Questionnaire and the BAS/BIS scales. Participants then donned a heart rate monitor and completed a baseline DEQ. Participants watched the neutral-, anger-, or fear-inducing films and completed the DEQ and RPE. Next, the neutral-, anger-, or fear-inducing music was started, and participants warmed up at a slow speed (2.5 MPH) for 5 min. Participants completed a 2-mile time trial, during which they completed the DEQ and RPE at mile 1. Participants then cooled down at 2.5 MPH for 5 min and completed the DEQ and RPE. The music was stopped, and participants sat quietly for a 15-min recovery period, during which time they had no access to distractions such as technology or reading material. Following recovery, participants completed a final DEQ and RPE, and watched a short positive film intended to reverse any negative emotions induced by the films and music (see Figure 1 for schematic representation of the study schedule). Throughout the test sessions, heart rate data were collected using Polar telemetry (Polar RS800CX). The three test sessions were spaced at least 1 week apart and were identical except for the motivational state induction. Following the third test session, participants were fully debriefed and compensated for their participation.

To reduce diurnal variation in cognitive and physical performance, test sessions were scheduled at the approximate same time of day within participants (±1 h). To reduce the influence of hydration status on physical performance, participants were asked to consume ½ L of water the night before a test session, and ½ L of water the morning of a test session. Participants were also required to consume at least one meal prior to a morning test session (i.e., breakfast) and at least two meals prior to an afternoon test session (i.e., breakfast, lunch). They were asked to abstain from alcohol intake for 24 h prior to the experiment.

#### Statistical Methods

Discrete Emotions Questionnaire, RPE, heart rate, and 2-mile run time were analyzed using repeated measures ANOVAs with motivational state induction (Neutral, Anger, Fear) and, where appropriate, Time (Pre-Induction, Post-Induction, Mile 1, Post-Cool-Down, Post-Recovery) as within-participant factors.

### Results

#### Two-Mile Run Time

**FIGURE 2:**
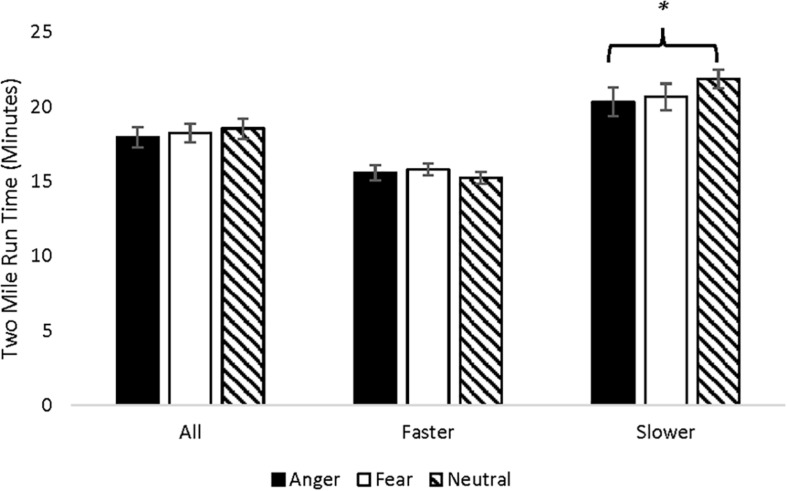
Two mile run time means (SEM) for each motivational state induction and running speed.

Across all participants, the 2-mile run time did not differ across motivational state induction (*p* = 0.331). In order to determine whether motivation states differentially influenced faster versus slower runners (Baldari et al., 2010), the 2-mile run times during the neutral condition were divided into fast and slow using the median splits method (the median cutoff point was 17:43 min), resulting in 17 participants (seven women, 10 men) with fast run times and 17 participants (12 women, five men) with slow run times. Among the faster participants, the 2-mile run time did not differ across motivational state induction (*p* = 0.448). Among slower participants, the anger induction reduced the 2-mile run time relative to the neutral induction, but did not differ from the fear induction, *F*(2,32) = 3.786, *p* = 0.033 (η^2^ = 0.191); see Figure 2.

#### RPE

Rated Perceived Exertion was greater at mile 1 and upon completion of the cool-down than before the warm-up, but did not differ between before the warm-up and after the 15-min recovery period, *F*(3,99) = 104.130, *p* < 0.001 (η^2^ = 0.661). RPE did not differ as a function of motivation state induction or motivational state induction by time (*p*’s > 0.33). RPE did not differ between the faster and slower participants (all *p-*values > 0.38).

#### DEQ

Analysis of self-reported anger showed main effects of motivational state induction, *F*(2,66) = 30.664, *p* < 0.001 (η^2^ = 0.135), and time, *F*(4,132) = 24.897, *p* < 0.001 (η^2^ = 0.105), which were qualified by a motivational state induction by time interaction, *F*(8,264) = 27.920, *p* < 0.001 (η^2^ = 0.218); see Table 4. Follow-up tests showed that self-reported anger was greater during the anger than neutral or fear induction immediately following the films, *F*(2,66) = 79.391, *p* < 0.001 (η^2^ = 0.706), and at mile 1, *F*(2,66) = 8.035, *p* = 0.001 (η^2^ = 0.040), and self-reported anger was greater during the anger than fear induction upon completion of the cool-down, *F*(2,66) = 3.735, *p* = 0.029 (η^2^ = 0.030). Self-reported anger did not differ across motivational state inductions before inductions or after the 15-min recovery (*p*’s > 0.29).

**TABLE 4:** Study 2 self-reported anger and fear (*n* = 34).

		Self-reported anger				Self-reported fear			
		Mean	SEM	Min	Max	Mean	SEM	Min	Max
Anger induction	Pre-induction	1.08	0.04	1.00	2.00	1.02	0.01	1.00	1.25
	Post-induction	2.85	0.18	1.00	4.75	1.48	0.13	1.00	4.00
	Mile 1	1.65	0.13	1.00	3.75	1.35	0.14	1.00	4.50
	Post-cool-down	1.52	0.17	1.00	4.75	1.33	0.13	1.00	4.00
	Post-recovery	1.15	0.08	1.00	3.50	1.17	0.12	1.00	4.75
Fear induction	Pre-induction	1.17	0.10	1.00	4.00	1.08	0.04	1.00	2.25
	Post-induction	1.11	0.07	1.00	3.00	3.16	0.28	1.00	6.00
	Mile 1	1.21	0.07	1.00	2.50	1.38	0.12	1.00	3.50
	Post-cool-down	1.13	0.06	1.00	2.25	1.40	0.16	1.00	4.75
	Post-recovery	1.04	0.02	1.00	1.75	1.10	0.07	1.00	3.25
Neutral induction	Pre-induction	1.05	0.04	1.00	2.00	1.07	0.04	1.00	2.00
	Post-induction	1.08	0.04	1.00	2.00	1.20	0.11	1.00	3.75
	Mile 1	1.26	0.08	1.00	2.50	1.22	0.07	1.00	2.50
	Post-cool-down	1.20	0.08	1.00	2.75	1.08	0.05	1.00	2.50
	Post-recovery	1.15	0.07	1.00	2.75	1.03	0.03	1.00	2.00

*SEM, standard error of the mean.*

Analysis of self-reported fear showed main effects of motivational state induction, *F*(2,66) = 15.415, *p* < 0.001 (η^2^ = 0.072), and time, *F*(4,132) = 34.274, *p* < 0.001 (η^2^ = 0.166), which were qualified by a motivational state induction by time interaction, *F*(8,264) = 22.025, *p* < 0.001 (η^2^ = 0.179). Follow-up tests showed that self-reported fear was greater during the fear than neutral or anger induction immediately following the films, *F*(2,66) = 32.325, *p* < 0.001 (η^2^ = 0.780), but not other time points (*p*’s > 0.06).

#### HR

HR was missing for two participants due to the dropped signal by the HR monitor. HR was lower during the motivational state induction than all subsequent time points, and higher during the run than all other time points, *F*(4,124) = 885.496, *p* < 0.001 (η^2^ = 0.919). HR did not differ as a function of motivation state induction or motivational state induction by time (*p*’s > 0.11).

### Discussion

Study 2 examined whether experimentally induced approach- and avoidance-oriented motivational states would influence running performance on a 2-mile time trial. All participants were regular exercisers, who scored at least 38 on the Godin Leisure Time Questionnaire, where a score of 24 is considered “active” (Amireault and Godin, 2015). They watched films validated in Study 1 to induce fear, anger, and neutral emotions, and then ran 2 miles as fast as possible while listening to music validated to induce the same emotions.

Results revealed that anger enhanced 2-mile time trial performance among participants whose 2-mile run times fell above (i.e., were slower than) the median during the neutral condition. Anger or fear did not influence the 2-mile time trial performance among participants whose times fell below (i.e., were faster than) the median or the sample as a whole. The results support previous research that music intended to increase emotional arousal enhanced aerobic and anaerobic exercise performance (Hall and Erickson, 1995; Karageorghis et al., 1996; Eliakim et al., 2007; Jarraya et al., 2012). The results also support the findings that less-active individuals experienced longer times to exhaustion when they listened to fast-tempo music than no music, whereas more-active individuals experienced no such effect of music (Baldari et al., 2010). The results extend this research by suggesting that the arousal component of emotions cannot entirely account for the results, as both our anger and fear inductions were characterized as high arousal, negative valence emotions. Our findings suggest that motivational states elicited may differentiate emotional states regarding their effects on athletic performance in certain individuals, but that there is no clear effect of the two motivational state components on 2-mile run times. This finding complements the work done with single-joint and gross motor behavior, and extends it to the domain of whole-body aerobic exercise, a behavior that characterizes many people’s daily lives. The results may also support research showing that viewing a competitive sprint cycling task, the Wingate test, as a challenge enhanced performance relative to viewing it as a threat (Wood et al., 2018). However, the findings should be interpreted with caution, as we did not find effects of anger across the sample as a whole, and did not include a measure of physical fitness such as maximum oxygen update (VO_2max_) to differentiate participants on the basis of aerobic capacity.

Rated perceived exertion did not differ between motivational state conditions or between faster and slower participants. Previous research has suggested that certain emotions, such as viewing positive relative to neutral pictures, reduced perceived exertion during exercise (Schmidt et al., 2009). Research comparing listening to music to no music has also found reduced perceived exertion across a range of music genres and exercise intensities (Szmedra and Bacharach, 1998; Potteiger et al., 2000; Yamashita et al., 2006). Thus, it is possible that listening to any music, regardless of the emotional or motivational state it engenders, benefits exertion responses to exercise.

Self-reported emotion showed that participants felt more anger than fear throughout the run and cool-down during the anger induction. However, their feelings of fear relative to anger dissipated by the first mile of the run. Given that the fear induction persisted for at least 10 min relative to anger, and at least 30 min to neutral in Study 1, it is possible that the 2-mile time trial itself mitigated emotional responses to fear. Although, to our knowledge, no research has examined the effect of exercise on feelings of fear, the findings support research indicating that exercise reduces state anxiety (Petruzzello et al., 1991; Ensari et al., 2015). Research employing the Profile of Mood States has also found reductions in the anger-hostility subscale following exercise (Berger and Motl, 2000). The present research was not designed to test the influence of exercise on fear and anger states, but may provide preliminary evidence that exercise ameliorates emotions that elicit avoidance-oriented motivational states such as fear.

The emotion inductions elicited relatively low levels of anger and fear. Indeed, on a scale ranging from 1 (“Not at all”) to 7 (“An extreme amount”), participants’ self-reported anger averaged 3.5 immediately after watching the anger-inducing films, and their self-reported fear averaged 3.25 immediately after the fear-inducing films. Laboratory or real life events, such as embarking on a run frustrated over racial inequities or fearful for a family members’ health, that elicit these emotional-triggering motivational states to a greater degree may have great effects on athletic performance.

Together the findings suggest that approach-oriented motivational states such as those elicited by anger may improve running performance, but that the effects are thus far limited to certain individuals such as slower running, and thus unclear. The results have implications for the nature of motivational preparation prior to athletic events that will benefit the performance the most, as well as how emotion regulation strategies could be employed to switch motivational states to ones that are most beneficial to athletic performance.

## Data Availability Statement

The datasets for this article are not publicly available because the funding agency does not permit public release of data products. Requests to access the datasets should be directed to GE, grace.e.giles4.civ@mail.mil.

## Ethics Statement

The studies involving human participants were reviewed and approved by the United States Army Research, Development, and Engineering Command and the Tufts University Institutional Review Board. The patients/participants provided their written informed consent to participate in this study.

## Author Contributions

GG, EA, and TB contributed to the study concept. GG, CH, and GE completed the data preparation. GG wrote the first draft of the manuscript. All authors contributed to the data analysis, manuscript revision, and read and approved the submitted version.

## Conflict of Interest

The authors declare that the research was conducted in the absence of any commercial or financial relationships that could be construed as a potential conflict of interest.
